# Photonic Sensors in Chemical and Biological Applications

**DOI:** 10.3390/bios12111021

**Published:** 2022-11-15

**Authors:** Zigmas Balevičius

**Affiliations:** Faculty of Electronics, Vilnius Gediminas Technical University, 10223 Vilnius, Lithuania; zigmas.balevicius@vilniustech.lt

Biosensors are described as analytical devices in which biological substances are detected by using various physicochemical detection systems [1]. Biosensors usually integrate bio-receptors in the vicinity of the sensing surface (optical, electrical, electrochemical, and mechanical). The specific binding of the analyte of interest to the target bio-receptor produces physicochemical perturbations on the sensor’s surface, which are transformed into readable information [2]. Because different scientific communities have been involved in the development and studies of biosensors and biosensing systems, a rather common situation in which different descriptions are used for biosensors appear. Biologists and biochemists usually tend to describe biosensors as two or more biological substances that interact with each other, and the sensitivity of such a sensor concept is first of all related with the binding rate and strength of the formed biological complex [3,4,5]. Chemists and physicists, meanwhile, describe the biosensor device via the detection system that is used to detect the formation of biological complexes (it can be based on physical, chemical or physicochemical phenomena). In such descriptions, the sensitivity of the biosensors are decided by the physical or chemical method that is used for the detection [6,7,8].

This Special Issue titled “Photonic Sensors in Chemical and Biological Applications” is devoted for bio- and chemical sensors that are based on optical signal detection. Optical or photonic sensors in most cases detect the amount of photons that are incident to the detector. Optical sensors are based on photon-in-photon-out and, therefore, are non-destructive and can be applied at solid/liquid interfaces, which is very important for biosensing applications [9,10]. Moreover, the in situ monitoring of various biochemical processes and label-free advantages (no labelling of protein molecules required) make optical sensing an attractive tool for the analysis of bio-substances [11]. Various optical configuration schemes and detection systems were developed to improve the sensitivity of optical responses and the monitoring of biochemical processes [12]. However, some disadvantages with respect to optical methods applications always exist. For instance, optical biosensors most often are not a method for chemical identification; thus, the chemical or biological specificity of the sensor’s surface should be exceeded via functionalization by using biochemical protocols for modifying the sensing surface [13].

In recent years, nanophotonic technologies produced noticeable impacts on the development of advanced optical sensing and their miniaturization, functionality, and sensitivity [14,15,16]. Studies on the behavior of light–matter interactions at the nanoscale provide new possibilities for discovering advanced optical biosensors, where knowledge from optical and electrical engineering and the material sciences can be applied in fields such as biology, biotechnology, and biomedicine [17,18,19,20]. The most common nanophotonic-based optical sensors rely on silicon photonic technologies and plasmonics [21,22,23]. The physical principles of such optical sensors are based on the exploitation of the evanescent field as a sensing probe. Various types of optical sensors for chemical or biological detection employs silicon photonics such as those based on photonic crystals [24,25], whispering gallery modes [26], Bloch surface waves [27], Mach–Zender interferometers [28], photoluminescence [29], and many others. Other widely used sensing surfaces are metallic; commonly, the surfaces are gold or silver, but other metals such as titanium or aluminum are also used [30]. From this list, gold is most widely used because of the stability to environmental conditions, as it can be easily modifiedd by thiol groups forming self-assembled monolayers on the gold’s surface for further modifications. The presence of thin metal films or metallic nanostructures predicts the excitation of various plasmonic resonances that are widely used, even in commercially available surface plasmon resonance (SPR) biosensors [7]. Surface plasmon polaritons are the coherent oscillations of free electrons in the metal and photons of the incident light. The enhancement of electric field at the metal/dielectric interface in the form of evanescent waves is extremely sensitive to changes in the refractive index of the medium and are further transformed during drastic changes in optical responses, such as intensity, phase, the momentum of resonance, or the state of light polarization [6,31,32]. Various plasmonic modes such as surface plasmon polaritons, localized surface plasmons, Tamm plasmons, surface lattice resonances, and hybrid plasmonic modes were applied for optical sensing based on plasmonics [33,34,35,36,37]. For distinct plasmonic resonances, the extension of electric fields to the dielectric medium is different, and this leads to different sensitivity properties. Some studies even reported the possibilities of single molecule detection [6]. On the other hand, various optical schemes for the excitation of plasmonic modes restrict or open possibilities for miniaturization of such biosensors. For example, the Total Internal Reflection (TIR) configuration (with prism) is inconvenient for miniaturization; meanwhile, Tamm plasmons, LSPR, or SLR can be excited directly by incident light.

Photonic biosensors demonstrated their high detection sensitivity and capabilities in clinical diagnostics for small viruses and low molecular mass proteins at the nanoscale [38,39,40], and they also possess chemical aspects such as providing various detections of gases [41]. They are applied for the detection of antibodies in blood serum and the detection of DNA and RNA. The possibility to detect viruses opens promising perspectives in the diagnostics of respiratory viruses such as SARS-CoV-2. For instance, to develop a sensitive testing system for the detection of spike and nucleoproteins that are important for SARS-CoV-2 infection, it is essential to identify specific antibodies and to better understand their properties during the recognition of the corresponding virus proteins. Thus, the physicochemical parameters of binding kinetics between specific antibodies and nucleoproteins are of high importance [19]. As mentioned above, these aspects of photonic biosensors are more important for biochemists and the biologist community. Meanwhile, progression in developing technologies from laboratory prototypes to the design of integrated compact photonic sensors is closer to the topic of this Special Issue. Advanced smartphone technologies offer possibilities to combine plasmonic biosensors together [42,43]; moreover, complementary metal–oxide semiconductor (CMOS) technology has been used for readouts in smartphone cameras [44].

The technological progress in the manufacture of photonic nanostructures and thin films led to vast applications with respect to the manipulation of light, and this has become achievable in many areas of science and technology. The characterization of optical properties and the design of photonic structures with desired optical parameters open new possibilities in managing light in such structures; as a result, possibilities in developing optical sensors with enhanced sensitivity and selectivity also emerged. This Special Issue is devoted to all kinds of optical sensing applications based on photonic nanostructures and surfaces. The aim of this Special Issue is to present original research papers and review articles on the latest experimental and theoretical studies in the field of optical sensing based on photonic nanostructures and surfaces.
